# Incidence of Co-Infections of HIV, Herpes Simplex Virus Type 2 and Syphilis in a Large Cohort of Men Who Have Sex with Men in Beijing, China

**DOI:** 10.1371/journal.pone.0147422

**Published:** 2016-01-28

**Authors:** Dongliang Li, Xueying Yang, Zheng Zhang, Zixin Wang, Xiao Qi, Yuhua Ruan, Yunhua Zhou, Chunrong Li, Fengji Luo, Joseph T. F. Lau

**Affiliations:** 1 Chaoyang Center for Disease Control and Prevention, Beijing, PR China; 2 Center for Health Behaviours Research, The Jockey Club School of Public Health and Primary Care, Faculty of Medicine, The Chinese University of Hong Kong SAR, Hong Kong, China; 3 State Key Laboratory for Infectious Disease Prevention and Control (SKLID), Collaborative Innovation Center for Diagnosis and Treatment of Infectious Diseases, Chinese Center for Disease Control and Prevention (China CDC), Beijing, China; 4 Department of Epidemiology and Health Statistics, School of Public Health, Soochow University, Suzhou, Jiangsu, China; University of Alabama at Birmingham, UNITED STATES

## Abstract

**Background:**

The HIV-epidemic among MSM in China has worsened. In this key population, prevalence of HSV-2 and syphilis infection and co-infection with HIV is high.

**Methods:**

A longitudinal study was conducted (n = 962) in Beijing, China, with three overlapping cohorts (n = 857, 757 and 760) consisting of MSM that were free from pairs of infections of concern (i.e. HIV-HSV-2, HIV-syphilis, HSV-2-syphilis) at baseline to estimate incidence of HIV, HSV-2, syphilis, and those of co-infection.

**Results:**

The incidence of HIV, HSV-2 and syphilis in the overall cohort was 3.90 (95% CI = 2.37, 5.43), 7.87 (95% CI = 5.74, 10.00) and 6.06 (95% CI = 4.18, 7.94) cases per 100 person-years (PYs), respectively. The incidence of HIV-HSV-2, HIV-Syphilis and HSV-2-Syphilis co-infections was 0.30 (95% CI = 0.29, 0.88), 1.02 (95% CI = 0.13, 2.17) and 1.41 (95% CI: 0.04, 2.78) cases per 100 PYs, respectively, in the three sub-cohorts constructed for this study.

**Conclusions:**

The incidence of HIV, HSV-2 and syphilis was very high and those of their co-infections were relatively high. Such co-infections have negative impacts on the HIV/STI epidemics. Prevention practices need to take such co-infections into account.

## Introduction

Prevalence of HIV and other sexually transmitted infections (STIs) among men who have sex with men (MSM) in China is very high. A meta-analysis and other previous studies reported HIV prevalence exceeding 10% in many parts of China [1–3]. Another review reported that prevalence of syphilis increased from 6.8% during 2003–2004 to 13.5% during 2007–2008 [4]. The prevalence of HSV-2 was 7.8% in Jiangsu province [5] and 24.7% in Chengdu city [6]. The incidence of HIV (2.6 to 5.4 per 100 person-years) [7–11], HSV-2 (5.92 per 100 person-years) [12] and syphilis (7.58 and 16.9 per 100 person years) [8,11] was extremely alarming [12].Therefore, the HSV-2 and syphilis epidemics pose a serious threat to that of HIV among MSM in China[13–15].

Coinfections between HIV and other STIs are common due to shared routes of sexual transmission. The incidence of syphilis among HIV-infected persons was 77 times greater than that of the general population [16]. In two multisite cross-sectional surveys targeting MSM in China, the prevalence of HIV-Syphilis and HIV-HSV-2 co-infections was 12.5% [17] and 3.2% [18], respectively, while such prevalence was 29.0%[19] and 9.1%[19] in Yunnan, respectively. HIV-HSV-2 and HIV-Syphilis co-infections resulted in poor treatment outcomes of such STIs [20].

HIV-HSV-2 co-infection increased transmissibility of HIV-1 and progression to AIDS [21]. Specifically, it has increased plasma HIV viral load [22–25] to a clinically significant level of 0.5 log10 copies/ml [26–27]. It has been associated with reduced HIV-specific CD8+ T cell responses and systemic immune activation [21]. Severity of symptomatic HSV-2 has shown a correlation with low CD4 counts [28]. HIV-HSV-2 co-infection has also caused genital ulcers [29] of extensive and persistent nature, which reoccurred frequently and showed atypical clinical presentations [30–32]. Several clinical trials investigated efficacy of using HSV-2 suppression as a strategy to prevent HIV transmission and to slow down HIV disease progression [26].

Syphilis causes ulcers and was associated with HIV acquisition [33, 34]. HIV-Syphilis co-infection was associated with a slight and transient decrease in CD4 cell count and an increase in viral loads [35]. *T*.*pallidum*co infection showed a deleterious impact on the immunologic and virologic status of HIV-infected person [36–37]. HIV infection may also increase clinical lesions and accelerate progression of syphilitic infection [38–39]. A review of clinical manifestations and treatment outcomes of syphilitic uveitis showed that treatment failures were more likely to occur to HIV positive patients than to HIV negative patients [40]. Individuals with CD4^+^ T-cell counts of less than 200 cells/μl were at higher risk of serologic non-response to syphilis [41].

There are prevalence studies but not incidence studies investigating co-infections between HIV-HSV-2 and HIV-Syphilis among MSM. Incidence of co-infection of STIs does not necessarily mean that two types of STIs were contracted at the same time point (e.g. episode of sexual intercourse). Instead, both infections occurred during the same follow-up period. In this descriptive 6-month cohort study, we reported the incidence of HSV-2-HIV, HIV-Syphilis and HSV-2-Syphilis co-infections among MSM in Beijing, China.

## Subjects and Methods

### Study design and study population

A longitudinal study was conducted among MSM in Beijing, China, during August 2009 to October 2010. Inclusion criteria were: 1) men of at least 18 years old, 2) self-reported having had anal or oral sex with at least one man in the last six months, and 3) currently living in Beijing. Exclusion criteria included: 1) no contact information provided and 2) planned to relocate from Beijing within the next six months.

Multiple methods were used to recruit study participants, including advertisements posted by a non-governmental organization working on HIV prevention on gay-friendly websites, dissemination flyers at gay-friendly venues (e.g. MSM clubs, bars, parks and bathhouses), and referrals made by participants. Participants were briefed about the study when they visited the HIV testing clinic of the Beijing Jingcheng Venereal Hospital. With written informed consent, risk-reduction counseling and face-to-face interviews were conducted by trained staff in a private room at baseline and 6-month follow-up visit. Experienced physicians from the Institute of STD/AIDS Prevention and Treatment in Beijing, China, then conducted a clinical examination and collected blood samples for HIV, HSV-2 and syphilis testing. To ensure anonymity, participants presented their pre-assigned identification code when they obtained their test results after one week. Those who were tested positive for HSV-2, HIV or syphilis were referred to clinics or hospitals to receive appropriate treatment and follow-up services. At each visit, participants received 50 RMB (approximately US$ 7.40) in cash, 12 free condoms and one free pack of lubricant. The research protocol was approved by the Institutional Review Boards of the National Center for AIDS/STD Control and Prevention of the China Center for Disease Control and Prevention.

**Fig 1:**
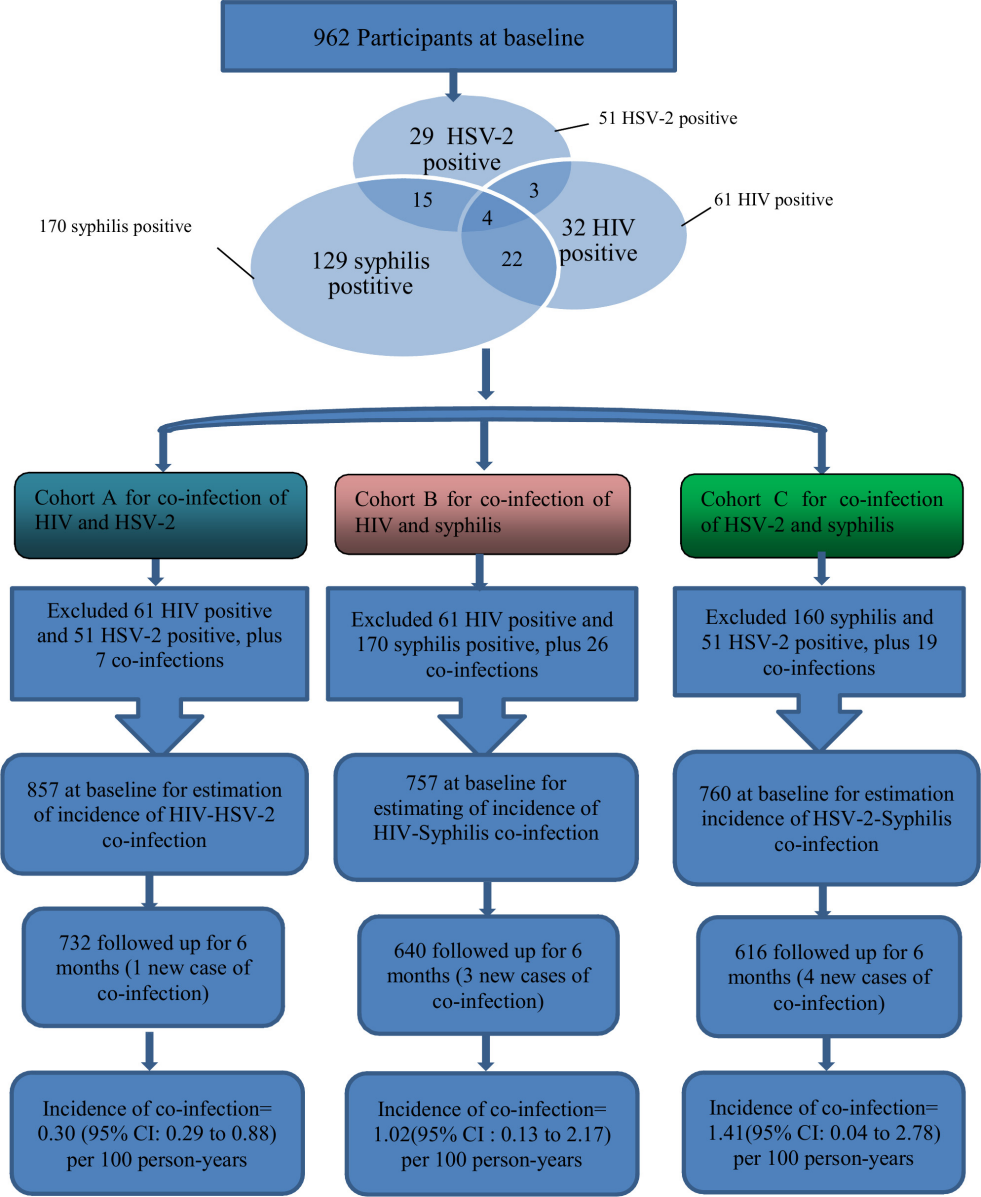
Processes for constructing the three cohorts for estimation of incidence of co-infections (HIV-HSV-2, HIV-Syphilis, HSV-2-Syphilis).

**Fig 2:**
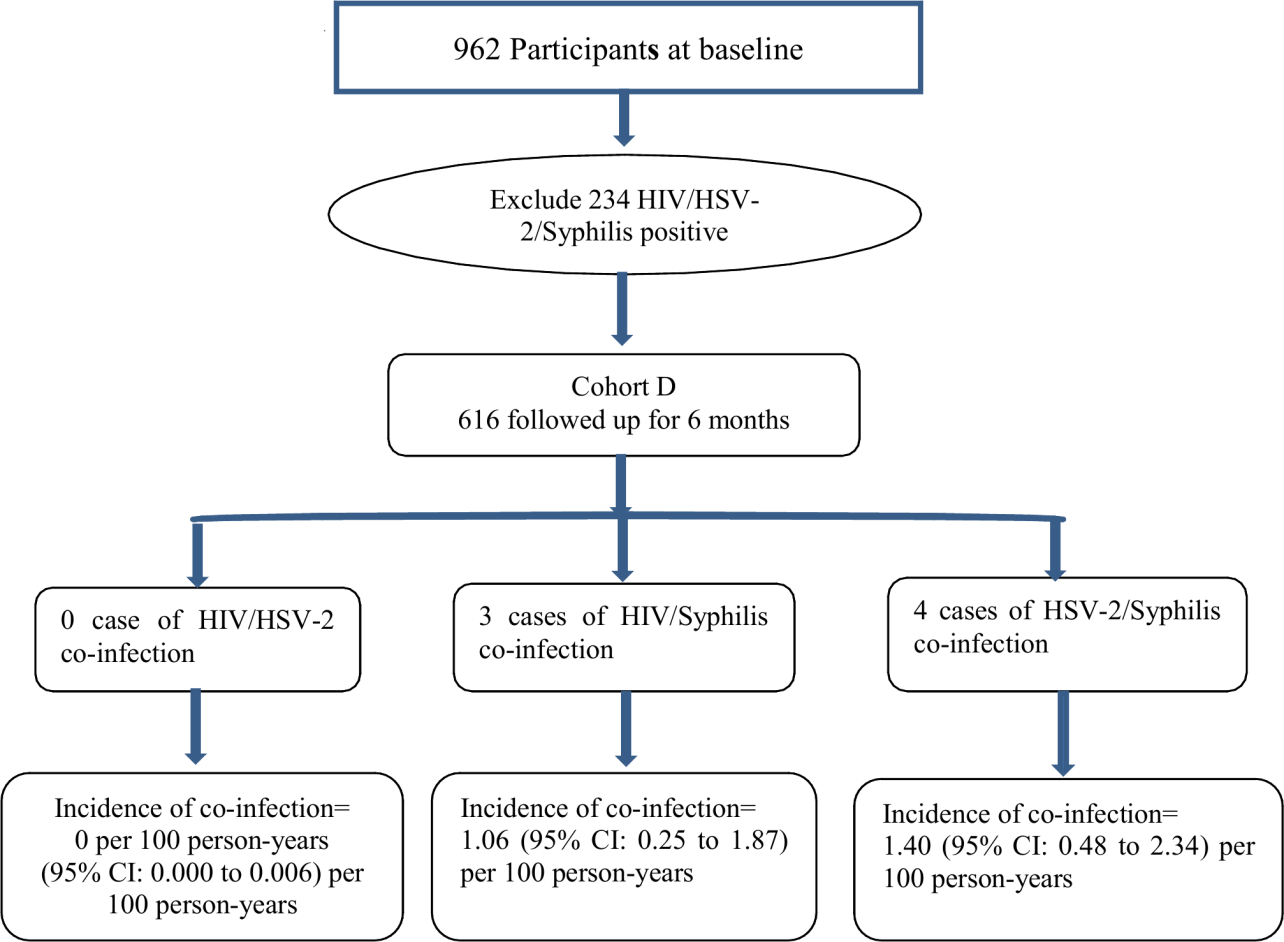
Processes for constructing the fourth cohort for estimation of incidence of co-infections (HIV-HSV-2, HIV-Syphilis, HSV-2-Syphilis, excluding all three infections at baseline).

An overall cohort of 962 participants were interviewed at baseline. Their prevalence of HIV, HSV-2 and syphilis was 6.3% (61/962), 5.3% (51/962) and 17.7% (170/962), respectively. They were divided into three overlapping sub-cohorts to investigate incidence of co-infection (i.e. HIV-HSV-2, HIV-Syphilis, HSV-2-Syphilis co-infections). At baseline, members were free from both infections of concern (e.g. free from HIV and HSV-2 for the cohort estimating incidence of HIV-HSV-2 co-infection). The effective cohort sizes were 857 (HIV-HSV-2), 757 (HIV-Syphilis) and 760 (HSV-2-Syphilis), respectively. The process for constructing these three cohorts has been explained in Fig 1. To provide another perspective, a fourth sub-cohort was constructed by excluding all HIV, HSV-2 and syphilis cases detected at baseline (n = 728) (Fig 2).

### Laboratory tests

Serostatus of HSV-2 was determined by using ELISA (Trinity Laboratories, San Antonio, TX, USA). HIV serostatus screening and confirmation were determined by using ELISA (InTec Products Company, Xiamen, China) and HIV-1/2 western blot confirmation (HIV Blot 2.2 WBTM, Genelabs Diagnostics, Singapore), respectively. Syphilis serostatus screening and confirmation were determined by using rapid plasma reagin (RPR) test (Shanghai Rongsheng, Shanghai, China) and the Treponemapallidum particle assay (TPPA) test (FujirebioInc, Tokyo, Japan), respectively.

### Statistical analysis

The incidence of HIV, HSV-2 and syphilis was estimated by using the number of sero-conversion detected within the 6-month follow-up period as the numerator and the cohort’s total number of person-year (PY) exposure to the risk of transmission as the dominator. For cases with sero-conversion, half of the follow-up duration (between the two visits) was used as their contribution to their total risk exposure [42]. The 95% confidence interval (CI) of the incidence was estimated by using Cox regression method. The Statistical Analysis System (SAS V.9.1 for Windows; SAS Institute Inc., Cary, NC, USA) software was used for data analysis.

## Results

### Baseline background characteristics

A summary is presented in Table 1. The three sub-cohorts had overlapping memberships and hence similar socio-demographic characteristics. Majority were under 30 years old (67.1%-69.5%), did not have Beijing permanent residency or Hu Kou (83.1%-84.0%), currently single (83.5%-84.2%), and of Han ethnicity (92.9%-93.3%), while 50.6%-52.5% had attended college or university and 58.2%-58.8% had had an average monthly income higher than about US$300. About two-thirds (65.5%-66.5%) self-identified himself as a homosexual person. Loss-to-follow-up status (about 20%) was associated with not being currently married (*p* = 0.011), less educated (*p*<0.0001), and not having Beijing permanent residency (*p*<0.0001) (Table 2).

**Table 1:** Background characteristic of members of the three cohorts constructed for estimating incidence of co-infection.

Characteristics		Cohort of HIV and HSV-2 negative MSM (N = 857) n (%)	Cohort of HIV and syphilis negative MSM (N = 757) n (%)	Cohort of syphilis and HSV-2 negative MSM (N = 760) n (%)
Age group (year)	<30	575(67.1)	515(68.0)	528(69.5)
	≥30	282(32.9)	242(32.0)	232(30.5)
Ever married	Yes	141(16.5)	122(16.1)	120(15.8)
	No	716(83.5)	635(83.9)	640(84.2)
Registered Beijing resident	No	713(83.2)	629(83.1)	638(84.0)
	Yes	144(16.8)	128(16.9)	122(16.0)
Ethnicity	Han	796(92.9)	705(93.1)	709(93.3)
	Minority	61(7.1)	52(6.9)	51(6.7)
Junior college or higher	No	423(49.4)	364(48.1)	361(47.5)
	Yes	434(50.6)	393(51.9)	399(52.5)
Monthly income, USD	≤300	358(41.8)	314(41.5)	313(41.2)
	>300	499(58.2)	443(58.5)	447(58.8)
Self-identified sex orientation	Not homosexual	287(33.5)	258(34.1)	262(34.5)
	Homosexual	570(66.5)	499(65.9)	498(65.5)

**Table 2:** Associations of the socio-demographic characteristic with loss of follow-up among MSM in Beijing, China, using univariate logistic regression model (N = 962).

Characteristics		Loss-to-follow-up N (%)	OR(95%CI)	*P*
Age, yr	<30	129(20.38)	1	
	≥30	64(19.45)	0.94(0.68,1.32)	0.7336
Ever married	Yes	20(12.66)	1	
	No	173(21.52)	1.89(1.15,3.11)	***0*.*011***
Beijing resident	Yes	9(5.73)	1	
	No	184(22.86)	4.87(2.44,9.74)	***<0*.*0001***
Ethnicity	Han	184(20.54)	1	
	Minority	9(13.64)	0.61(0.30,1.26)	0.1768
Junior college or higher	Yes	67(14.23)	1	
	No	126(25.66)	2.08(1.50,2.89)	***<0*.*0001***
Monthly income, USD	≤300	80(20.30)	1	
	>300	113(19.89)	0.97(0.71,1.34)	0.8759

### Incidence of HIV, HSV-2 and syphilis

In the overall cohort (n = 962), the incidence of HIV, HSV-2 and syphilis was 3.90 (95% CI = 2.37, 5.43), 7.87 (95% CI = 5.74, 10.00) and 6.06 (95% CI = 4.18, 7.94) per 100 PYs, respectively.

### Incidence of co-infection of HIV-HSV-2, HIV-Syphilis and HSV-2-Syphilis

In the HIV-HSV-2 sub-cohort, 14 and 30 new HIV and HSV-2 cases, respectively, were detected during the 6-month follow-up period. There was one case of co-infection; the incidence of HIV-HSV-2 co-infection was hence 0.30 per 100 PYs (95% CI = 0.29, 0.88).

In the HIV-Syphilis sub-cohort, 15 and 17 new HIV and syphilis cases were detected during the follow-up period, respectively. There were three cases of co-infection; the incidence of HIV-Syphilis co-infection was hence 1.02 per 100 PYs (95% CI = 0.13, 2.17).

In the HSV-2-Syphilis sub-cohort, 17 and 22 new syphilis and HSV-2 cases, respectively, were detected during the follow-up period. There were four cases of co-infection; the incidence of HSV-2-Syphilis co-infection was hence 1.41 per 100 PYs (95% CI: 0.04, 2.78).

Comparing incidence of co-infections obtained from the fourth sub-cohort (which removed all HIV, HSV-2 and syphilis cases at baseline) with those of the three afore-mentioned sub-cohorts (which only removed the two infections of concern at baseline), the incidence of HIV-Syphilis co-infection (changed from 1.02 to 1.06) and HSV-2-Syphilis co-infection (changed from 1.41 to 1.40) remained almost the same, while that of HIV-HSV-2 changed from .3 to 0.

## Discussion

Our findings reinstated that the HIV/STIs epidemics are very severe among MSM in Beijing, China. Previous studies have consistently reported high prevalence of HIV, HSV-2 and syphilis targeting this population[12,18, 43–44]. In corroboration we found an alarmingly high incidence of HIV, HSV-2 and syphilis, as well as noticeable incidence of pair-wise co-infections among these three infections. In particular, the incidence of HSV-2-Syphilis co-infection (1.41 per 100 PYs) was higher than that of HIV-HSV-2 (.3 per 100 PYs) and HIV-Syphilis co-infections (1.02 per 100 PYs). It is because HSV-2 and syphilis are the most common types of genital ulcer diseases [45]. Furthermore, previous studies showed that the pooled prevalence of HIV-Syphilis co-infection had increased substantially from 1.4% during 2005–2006 to 2.7% during 2007–2008, and kept increasing as an annual rate of 0.5% across the country [4]. The relatively high incidence of HIV-Syphilis co-infection implies re-emergence of a HIV-Syphilis co-epidemic among MSM in China, which might have partially contributed to the recently observed high HIV incidence.

No study has reported whether those with HSV-2-Syphilis co-infection would lead to even higher risk of contracting HIV than those with mono-infection of HSV-2 or syphilis. If that is true, the relatively high incidence of HSV-2-Syphilis co-infection may explain partially the high incidence of HIV among MSM in China. Such needs to be investigated. Better integration of treatment and prevention of HIV and other STIs is warranted.

HIV interventions should also disseminate messages about other STIs to MSM in China, such as those related to clinical consequences of co-infections for HIV-HSV-2 and HIV-Syphilis. For instance, HIV negative MSM and those with HSV-2 or syphilis infection should be fully informed that HSV-2 and syphilis can facilitate co-infections with HIV [45]. HIV positive MSM without HSV-2 and syphilis infection should also be informed that new co-infections with HSV-2 and syphilis can have adverse effects on disease progression and mortality. Perceived severity of diseases, according to the Health Belief Model (HBM), can promote preventive behaviors [46]. Some MSM do not consider syphilis to be a serious disease as it is treatable and curable [47]. Low perceived severity may also apply to HSV-2 as it is not fatal [48]. Information about consequences of co-infections may increase perceived severity of these STIs and reduce UAI among MSM of both HIV positive and negative sero-status.

Risk perceptions on genital warts and syphilis were negatively associated with UAI [9, 49, 50]. Perceived susceptibility is another construct of the commonly used HBM [50]. Perceived susceptibility in contracting syphilis or HSV-2 together with HIV should be promoted among MSM in China. Such health promotion can be based on our data. We found that the incidence of HIV and HIV/HSV-2 co-infection were 3.90 and .3 per 100 PYs, respectively, implying that out of 100 MSM, 3.9 would contract HIV and .3 would contract both infections in one PY, i.e., one out of 13 (.3/3.90) new HIV infections would be a new HIV-HSV-2 co-infection. Likewise, we estimated from the data of the HIV-Syphilis sub-cohort that one out of four new HIV infections would also be a new HIV-Syphilis co-infection (1.02/3.90), as the two incidence of concern were 1.02 and 3.90, respectively. Similarly, about one out of six new syphilis infections would be a new HIV-Syphilis co-infection (1.02/6.06). The observed co-infections did not necessarily occur at the same time point (i.e., episode of sexual intercourse), although that could not be ruled out. We are, however, certain that the two infections occurred within six months (the follow-up period). Sequential infections occurring within a relatively short time period reflect continuous practice of sexual risk behaviors (e.g. UAI) among MSM. Therefore, MSM should be reminded that the chances of them contracting HIV ‘shortly’ after having newly contracted other types of STIs (e.g. syphilis) are quite high. The same can be said for contracting HSV-2 or syphilis ‘shortly’ after having newly contracted HIV. The chances of contracting HSV-2 after contracting syphilis and vice versa would even be higher. Such information about sequential new infections of HIV/STIs may increase risk perceptions and perceived susceptibility of these diseases and reduce UAI among MSM in China.

HSV-2 and syphilis testing should therefore be provided alongside with HIV testing. MSM with risk behaviors need to be persuaded to take up testing for multiple types of STIs on a regular basis. They, and in particular those found positive in one type of STI, should be reminded seriously about the high risk of subsequent co-infection(s) that could occur within a few months. An especially important message is that one has a high likelihood of contracting HIV in the next few months if they were newly tested positive with syphilis (about one out of six). Those tested positive for these diseases should be provided with counseling and treatment. Such advice on risks may increase perceived susceptibility of contracting HIV among MSM with newly detected syphilis and HSV-2 infections.

The findings also have implications for service providers. HIV workers should be made aware of the findings and their implications in order to promote regular and multiple types of HIV/STIs testing and reduction of risk behaviors among MSM, especially for those tested positive in syphilis. There are criticisms that HIV and STI prevention and treatment centers in China are not integrated enough [10, 51]. Establishment of integrated gay-friendly clinics for comprehensive testing, treatment and prevention for both HIV and STIs is an important step for tackling issues of high incidence of co-infections. Previous studies also showed that MSM sometimes did not seek treatment for their STIs [52,53]. Clinicians should be made aware that there is a high chance to detect new co-infected HIV/syphilis/HSV-2 cases among those with mono-infection of such STIs. The presence of co-infections between HIV and HSV-2/syphilis may lead to worse clinical outcomes among HIV positive MSM, creating difficulties in clinical management.

Although numerous studies have reported high prevalence of HIV, HSV-2 and syphilis among MSM in China, this is the first longitudinal study reporting incidence of co-infections of HIV-HSV-2, HSV-2-Syphilis and HIV-Syphilis simultaneously in a relatively large cohort of MSM in China. As this is the first study of the type, clinical and management implications for co-infections newly found within a relatively short time period among those with mono-infection (e.g. a newly detected HIV positive case contract syphilis or HSV-2 after a few months), as compared to similar co-infections jointly found at first sight (e.g. a person found both HIV and syphilis positive at the same time point) and those who had contracted a disease for some time and contracting a new one (e.g. someone had been diagnosed as HIV positive for many years and now contracted syphilis in addition), were unknown and requires further research. Future studies should also look at related co-infections in MSM population in other countries as well as other types of co-infection of STIs.

The HIV-HSV-2, HIV-Syphilis and HSV-2-Syphilis sub-cohorts were free from the two infections of that cohort but might include cases having the third type of infection of concern. For instance, the HSV-2-Syphilis cohort included HIV positive cases, while HIV positive serostatus may also increase risk of HSV-2 and syphilis infection. Thus HIV positive and negative participants may have a different incidence of HSV-2-Syphilis co-infection. However, separate estimations were not feasible due to the limited sample size. We created a fourth sub-cohort which eliminated all HIV/HSV-2/syphilis cases found at baseline. The incidence of co-infection found in that sub-cohort was similar for HSV-2-Syphilis and HIV-Syphilis sub-cohorts. However, the HIV-HSV-2 co-infection changed from .3 to 0 per 100 PYs, as there was only one co-infection case and it was removed from analysis because he was also syphilis positive at baseline. The overall picture remained the same that incidence of HIV-HSV-2 co-infection would be lower than those of HIV-Syphilis co-infection, suggesting that newly diagnosed HIV cases are more likely to have co-infection with syphilis than with HSV-2.

Despite some strength, the study has a number of limitations. First, sample selection bias may exist as it was not based on a random sample; there were also loss-to-follow-ups and some of their characteristics (e.g. education level) were different from those remained in the study. Hence, the sample may not be representative of MSM in Beijing. Many participants did not have Hu Kou (permanent residency) in Beijing as there were internal migrants; they do not enjoy some social welfare services but can live normal lives and work in Beijing. Only 38% of the people living in Beijing have permanent residency. Furthermore, we have excluded those with no intention to stay in Beijing in the next six months. Most of the published studies targeting MSM in China include those both with and without permanent residency [12,54–55]. Second, as we only studied MSM in Beijing, generalization of the findings to other cities in China or the entire nation requires caution. Third, the cohort size, although relatively large, could only detect a small number of incident cases of HIV, HSV-2 and syphilis; a small change in the numerator could result in a sizable change in incidence estimation although changes in 95% CI would be relatively small. Due to the small number of new infections, we could not estimate factors of co-infections. Fourth, a six month period of follow up is relatively short to detect more new co-infections.

In sum, we estimated the incidence of three types of co-infections for the first time. Although prevalence of HIV, HSV-2 and syphilis is high among MSM in China, majority of them still be negative in one, two or all three of these infections. They hence have a similar infection status as our participants and fit into the definitions of the fourth sub-cohorts of this study. Our findings on incidence of co-infections are hence applicable to reality. As discussed, the findings can be translated into potentially useful health communication messages for HIV/STIs prevention. We however, do not know about clinical implications of the observed co-infections that occurred within a short period of time onto disease progression and clinical outcomes. Future investigations are warranted.
